# The College Nominations

**Published:** 1921-03-19

**Authors:** 


### The College Nominations

The general apathy with which members of the College have hitherto regarded the ballot-papers which decide the elections to the Council is due, as everyone now recognises, to the haphazard method of open nomination. Under this system a long unenlightening list of names is submitted to the voter, who, in despair, adopts one of two courses, either throwing the paper into the waste-paper basket, or making chance shots at any name she happens to be acquainted with.

Schemes for making the voting-paper more comprehensible to the ordinary College member have been under discussion for some time past. To reform the nomination system by throwing responsibility for nominations into the hands of the Centres instead of private members was the plan advocated in these columns, and it is being worked out with great skill in various directions. The problem is one of considerable difficulty, as an ideally elected Council must be representative not only of various nursing interests but also of different localities in England and Wales. Meetings have been held in most of the Centres to discuss the best way of presenting candidates for nomination. The Leeds Centre has nominated Miss Earle, the Sheffield Centre Miss Innes, and the Norwich Centre Miss Corder. It is understood that Miss Burgess, of Crumpsall Infirmary, will be nominated to represent Poor-law interests, though she will probably not be the only candidate to be put forward for this important work. So far all promises a far more live and interesting election.

The London Centre has not been behindhand in these activities, but it received an unexpected check in its forward policy at its meeting on February 28 (See The Hospital, March 5, page vi.). Some anxiety was apparently entertained as to the extent to which members might feel their individual freedom of action restrained by official nominations, and it was feared that the position of candidates nominated by private individuals might be prejudiced. The quality expected of a great organisation is prudence rather than enthusiasm in the conduct of its affairs. The College must study to be just even if the skies fall. The Executive Committee is largely composed of London members, and they are right to guard the influential section at headquarters from any imputation of packing the Council. These and other considerations pointed to the necessity for extreme caution. Hence the decision on February 28 to take no further steps to nominate official candidates. Subsequently, however, a further meeting was called for March 15 to reconsider the question, and after hearing a most lucid address from Mrs. Oliver Strachey on Election Organisations, the meeting reversed its former decision and by a substantial majority decided to take a postal ballot on the eight or nine candidates nominated by Centre members, with a view to reducing the number to be thus officially nominated to five, of whom three are to be London representatives and two provincial, the voting to be on a modified system of proportional representation. The further important decision was taken empowering the Executive Committee to co-operate with provincial centres to secure the election of its five official candidates and to offer reciprocal support for the candidates of other centres.

There can be no doubt that these are the lines on which to proceed to achieve democratic and fair representation of all interests. Zealous elections can only come about through diligent spade work right through the field, and this is now in full swing. The London Centre members have reason to be grateful to Mrs. Oliver Strachey for her wise counsel and her clear exposition of the points to be borne in mind. Meantime let the private nominator hold her hand before inducing distinguished members of the medical profession to allow themselves to be nominated. A glance at the table of attendances at Council meetings printed in the current number of the *Bulletin* will show that these over-burdened individuals, however sincerely they may intend to be present, can very rarely indeed put in an appearance at meetings. It involves the exclusion of zealous members of the Council to put on the voting-paper names so well known and revered as to be sure of adhesion, names, however, which yet as Councillors remain names only. Nomination is a very serious duty, to be undertaken only in consultation with a group of others. Much harm may be done by jotting down names without consideration, for such names only confuse the voters.

